# Presentation of laboratory test results in patient portals: influence of interface design on risk interpretation and visual search behaviour

**DOI:** 10.1186/s12911-018-0589-7

**Published:** 2018-02-12

**Authors:** Paolo Fraccaro, Markel Vigo, Panagiotis Balatsoukas, Sabine N. van der Veer, Lamiece Hassan, Richard Williams, Grahame Wood, Smeeta Sinha, Iain Buchan, Niels Peek

**Affiliations:** 1 NIHR Greater Manchester Primary Care Patient Safety Translational Research Centre, The University of Manchester, Manchester, UK; 2 Health eResearch Centre, Farr Institute for Health Informatics Research, London, UK; 3 Centre for Health Informatics, Division of Informatics, Imaging and Data Science, The University of Manchester, Manchester, UK; 4 Renal Clinic, Salford Royal NHS Trust, Salford, UK; 5 School of Computer Science, The University of Manchester, Manchester, UK; 6 Microsoft Healthcare, Microsoft Research, Cambridge, UK; 7 Centre for Health Informatics, City University, London, UK

**Keywords:** User-computer interface [MeSH], Computers/utilization [MeSH], Decision making [MeSH], Personal health record [MeSH], Patient access to records [MeSH], Patient portals, Decision support systems, clinical [MeSH], Personal health records, Patients access to records, Laboratory test results, Computer utilization, Eye tracking

## Abstract

**Background:**

Patient portals are considered valuable instruments for self-management of long term conditions, however, there are concerns over how patients might interpret and act on the clinical information they access. We hypothesized that visual cues improve patients’ abilities to correctly interpret laboratory test results presented through patient portals. We also assessed, by applying eye-tracking methods, the relationship between risk interpretation and visual search behaviour.

**Methods:**

We conducted a controlled study with 20 kidney transplant patients. Participants viewed three different graphical presentations in each of low, medium, and high risk clinical scenarios composed of results for 28 laboratory tests. After viewing each clinical scenario, patients were asked how they would have acted in real life if the results were their own, as a proxy of their risk interpretation. They could choose between: 1) Calling their doctor immediately (high interpreted risk); 2) Trying to arrange an appointment within the next 4 weeks (medium interpreted risk); 3) Waiting for the next appointment in 3 months (low interpreted risk). For each presentation, we assessed accuracy of patients’ risk interpretation, and employed eye tracking to assess and compare visual search behaviour.

**Results:**

Misinterpretation of risk was common, with 65% of participants underestimating the need for action across all presentations at least once. Participants found it particularly difficult to interpret medium risk clinical scenarios. Participants who consistently understood when action was needed showed a higher visual search efficiency, suggesting a better strategy to cope with information overload that helped them to focus on the laboratory tests most relevant to their condition.

**Conclusions:**

This study confirms patients’ difficulties in interpreting laboratories test results, with many patients underestimating the need for action, even when abnormal values were highlighted or grouped together. Our findings raise patient safety concerns and may limit the potential of patient portals to actively involve patients in their own healthcare.

**Electronic supplementary material:**

The online version of this article (10.1186/s12911-018-0589-7) contains supplementary material, which is available to authorized users.

## Background

Patient portals are frequently assumed to motivate and involve patients in their own health and care [1–6]. These systems allow patients to book appointments online, view laboratory test results, or communicate with their physicians. Currently, many patient portals are for patients living with long-term conditions [7, 8], where longitudinal follow-ups are complex, and where self-management is a key component [9].

Patients mainly use patient portals to check their laboratory test results [10–14]. Yet, laboratory test results are among the most difficult data for patients to understand [15–18], and there are concerns about the suitability of patient portals to support patients in this task [7, 19]. Misinterpretation of laboratory test results can have adverse effects on patient safety, increase patient anxiety, and reduce self-management efficacy [7, 14, 20]. With the increasing availability of patient portals [21, 22], it is therefore important to understand how patients interact with, and process, laboratory test results to inform the online presentation of this information for accurate risk interpretation and improved user interaction.

It is known that several contextual factors, like a patient’s numeracy and health literacy, can influence risk interpretation in the context of online laboratory test results [17, 23], however little is known about the effect of presentation on risk interpretation and interaction with this type of information. Previous studies have reported that patients found it difficult to understand laboratory test results shown in tables [15–17, 23] and graphs [15, 18], even when patients were familiar with the clinical scenarios at hand (i.e. glucose level monitoring for diabetes patients) [17]. A possible explanation for this phenomenon is the way numerical data were presented to patients [24]. Horizontal coloured bars that contextualise the latest value in relation to the standard range have been one of the few alternative presentations that have been implemented to improve risk interpretation of laboratory test results [16, 23]. They outperformed tables in terms of perceived usefulness [16] and decrease perceived urgency of borderline (i.e. low deviance from reference range) laboratory test results [23]. However, so far they have been only tested on static implementations (e.g., not on fully functioning patient portals) and on a limited number of laboratory tests at the same time. More widely, there are new presentation techniques that have been shown to enhance human decision making and information seeking behaviours in other web-based contexts [25–30]. However, these have not been tried in patient portals. For example, grouping (e.g., presenting together items that have similar characteristics) combined with the overview preview metaphor (e.g. where all user interaction happens within the same page) have been proven more effective than tables or list-based presentations in information-seeking and decision-making tasks [28–30]. However, this approach was never tested in the context of online laboratory test results.

We conducted a controlled study in which participants accessed different web-based presentations of laboratory test results across different clinical scenarios. We hypothesized that presentations using colour, which positively influences human cognition for risk interpretation tasks [31], and graphical cues (e.g., horizontal coloured bars and personalised grouping) improve patients’ interpretation of laboratory test results presented through patient portals. To investigate whether correct interpretations were associated with specific visual search behaviours, our secondary objective was to assess and compare metrics derived from eye-tracking data including search efficiency and cognitive load.

## Methods

### Study population

We focused on patients with Chronic Kidney Disease (CKD), for whom there is an online platform, named PatientView [32], that provides access to laboratory results and is available in 90% of renal units in the UK. We included patients who had a kidney transplant (at least 12 months before recruitment). These patients undergo longitudinal follow-up with quarterly visits, including a review of their laboratory test results. This allowed us to obtain a homogeneous group of participants in terms of their experience with and knowledge of the disease. We excluded patients with any visual impairment, to avoid ineffective eye tracking data collection, and patients who did not use the internet in their everyday lives, to ensure a certain level of digital proficiency required to be a potential patient portal user.

We recruited 20 patients from the Renal Transplant Clinic at Salford Royal NHS Foundation Trust (SRFT), which has one of the largest communities of PatientView users in the UK. The study received ethical approval from NHS and local R&D ethical committees (IRAS ID: 183,845). Research nurses from the NIHR Clinical Research Network Portfolio (Study CPMS ID: 20,645) were responsible for approaching eligible patients and collecting signed informed consents of patients willing to participate.

### Patient involvement

Increasingly patients are becoming involved as active partners in planning and undertaking research, rather than only as participants or data sources [33]. Throughout the project, we involved three patients from the local CKD patient community (http://gmkin.org.uk/) as collaborators, of whom two had experience of using PatientView. Initially, these patient collaborators participated in a workshop with researchers, during which they provided insights on their perceived importance and relevance of monitoring laboratory test results in CKD, and the role of PatientView in such tasks. During the workshop the patient collaborators also commented on preliminary visual presentations that we had prepared based on the literature discussed in the background and suggested additional features that might support them in interpreting their laboratory results on PatientView. After the workshop the patient collaborators were involved via email, providing comments on visual presentations and the study protocol. One patient collaborator also pilot-tested our data collection procedure and commented on our interpretation of the results.

The continuous involvement of patient collaborators allowed us to design a more realistic, relevant and acceptable experiment. Furthermore, two of our patient collaborators were experienced PatientView users, often interacting with other fellow patients in the local CKD patient community on this topic. Therefore, their advice and feedback was extremely important while developing our visual presentations.

### Controlled study design

The study followed a “3 × 3” repeated measures, within-subjects design where each participant used, in a random order, three different presentations of web-based laboratory test results to complete the same simulated task in three different clinical scenarios. These were designed by a nephrologist at SRFT to reflect:

- *High risk clinical scenarios:* characterised as life threatening situations that required immediate action; creatinine and estimated Glomerular Filtration Rate (eGFR) (i.e. the main indicators of kidney function [34]), as well as potassium (associated with higher mortality in kidney patients [35]) strongly deviated from the standard range.
- *Medium risk clinical scenarios:* identified by abnormal creatinine and eGFR, but normal potassium and stable conditions; no urgent action was necessary, however further tests were required within 4 weeks;
- *Low risk clinical scenarios:* characterised by normal creatinine, eGFR and potassium; not requiring any action until the next scheduled appointment.

In addition to creatinine, eGFR and potassium each scenario included 25 laboratory test results with different deviances from the standard range in relation to the reflected risk (i.e. higher risk scenarios had more concomitant abnormal values).

There was no previous training or time limit for performing the task. Participants could decide to stop exploring the laboratory test results whenever they felt ready to reply to the follow up questions.

The controlled study was conducted at the Interaction Analysis and Modelling laboratory of the University of Manchester, and each patient participated individually. All participants performed the tasks using a desktop computer with a 17-in. screen with an embedded eye tracker (Tobii T60), which permits a 60-Hz sampling rate, 0.5 degrees gaze point accuracy, and free head motion.

### Presentations

**Fig. 1:**
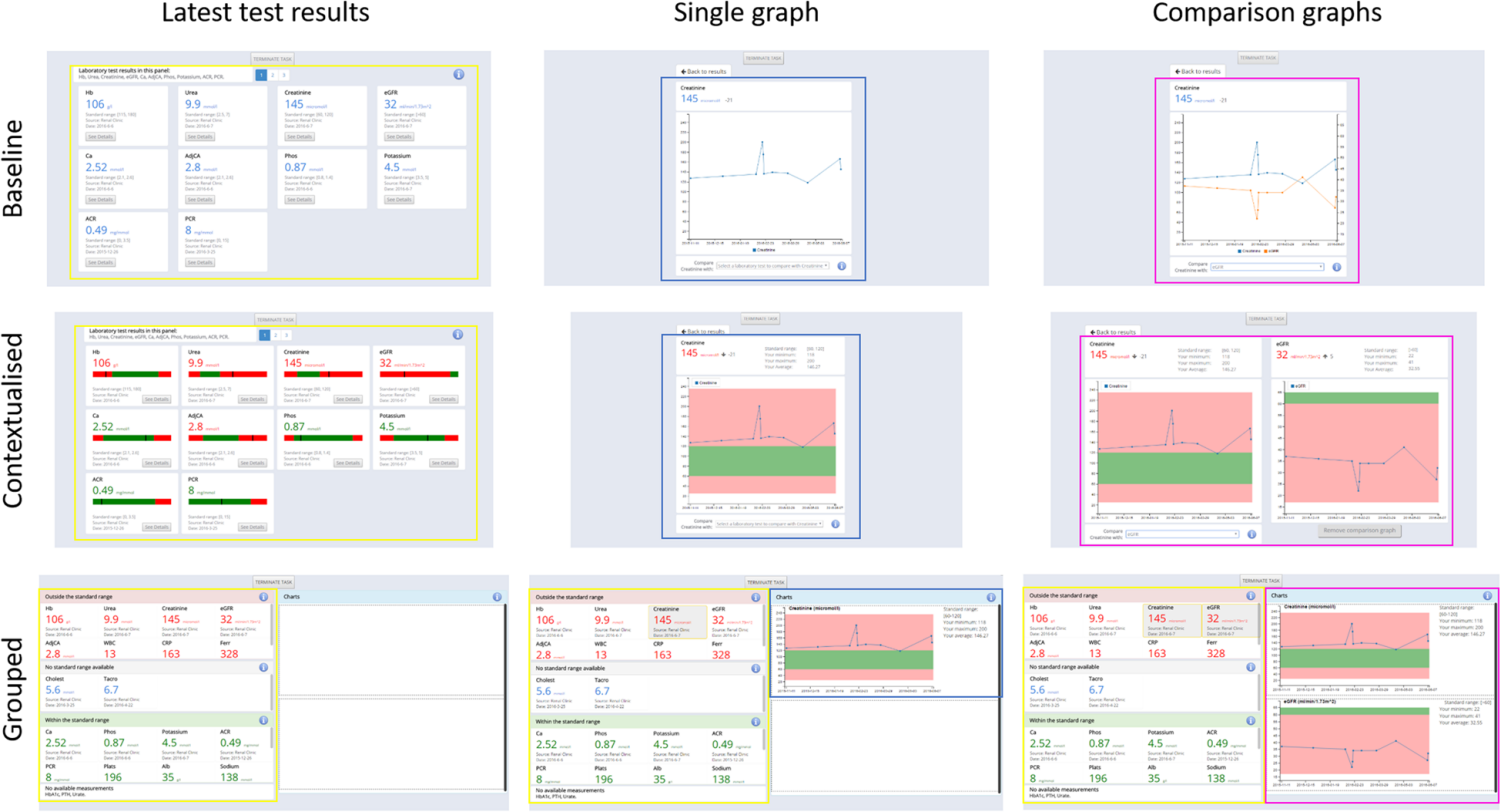
Latest test results overview and longitudinal detailed information for one or two tests at the same time across the three presentations. Coloured rectangles represent the different areas of interest (AoIs) we defined across the three presentations (yellow: latest test results; blue: single graph; purple: comparison graphs)

We implemented three presentations of laboratory test results (see Fig. 1 and Additional file 1: Figure S1-S9 for details). In developing these presentations, we aimed at maintaining similar amount of textual information, but showing it through different formats and visual cues to enhance patient interpretation. These were chosen based on review of the literature and patient collaborators’ feedback on preliminary prototypes.

The *Baseline presentation* was based directly on, and very similar to, the current PatientView [32] interface, which uses tiles to show the latest available laboratory test results. Each tile reports information on the value of a laboratory test, its unit, the date of the test, data source, and standard range. By clicking on a tile the user can access previous results (i.e. longitudinal information), with the possibility of comparing it with another test within the same graph. This feature can be particularly useful in the context of CKD, where the use of some medications improves renal function but can also negatively affect the functioning of other organs. This would be reflected by abnormal values in laboratory test results.

The comparison presentations were based on the Baseline presentation, but provided different visual cues, colours and tools to show normal and abnormal values. Chronic patients have an increased risk of having test results falling slightly outside the reference range, with only those with high deviance from the population reference range likely to be clinically relevant [36]. Therefore, the second presentation (*Contextualised presentation*) used horizontal coloured bars that contextualise the latest value in relation to the standard range in each tile [16, 23, 37], which, as already said in the background, have outperformed tables in terms of perceived usefulness [16] and decrease perceived urgency of borderline (i.e. low deviance from reference range) laboratory test results [23]. The third presentation (*Grouped presentation*) aimed at helping patients in identifying abnormal results, and made use of personalised grouping (i.e. dynamically grouping the tiles in “Outside the standard range”, “No standard range available”, and “Inside the standard range”) and the aforementioned overview-preview metaphor [28–30]. Both the Contextualised and Grouped presentation graphs reported personalised statistics for the selected test [38–40] and used colours, showing the area inside the standard range in green and the remaining area in red [41]. We applied the same approach to colour the latest laboratory test results in the tiles in both presentations. This had the aim of drawing patient’s attention on laboratory test results that might require more careful review (i.e. the ones in red), as well as visually filtering out those ones that were normal (i.e. the ones in green).

All plots within the same scenario displayed the same time period to avoid the well-known difficulties with scale changes [42]. Particularly, each plot displayed the period from the earliest to the latest laboratory test result available, which for each scenarios was between 1 and 2 years. Furthermore, in order to make the task as realistic as possible (i.e. pretending to have just received some results from the clinic), all results were shifted towards the day the experiment was carried out.

Since the purpose of the experiment was to expose patients to three very different types of scenario, keeping fixed ranges for each laboratory test as suggested by Zikmund-Fisher et al. [23] would have been detrimental for our purpose. That is, predefined value ranges specific to the type of clinical scenario (i.e. low, medium and high risk) might have revealed the pattern shown in the data. Therefore, we dynamically tailored the value ranges shown in the plots, for all three presentations, and horizontal coloured bars, for the Contextualised presentation. Specifically, the range of values shown always included the minimum and maximum value of the longitudinal series of results for each laboratory test. If the minimum or maximum fell inside the laboratory test reference range, they were replaced by lower and upper reference range limit, respectively. To ensure that the within and outside reference range areas were always clearly displayed, an offset was added to the minimum and maximum of the value range.

### Data collection

At the beginning of the experiment each participant was asked to complete four questionnaires: demographics (age, gender, education, years since transplant, frequency of internet usage, and frequency of PatientView use); Subjective Numeracy Scale (SNC) [43] on a 1–6 scale; self-reported health literacy on a 0–4 scale based on Chew et al. [44], with lower scores indicating better health literacy; and graph literacy, calculated as % of correct answers on the questionnaire from Galesic et al. [45].

After exploring each presentation of laboratory test results, participants were asked to respond to a question about their behavioural intentions in relation to what they saw, which we used as a proxy of their risk interpretation. Particularly, patients were asked what they would do in real life if the results they had just explored were their own. They could choose between: 1) Calling their doctor immediately (high interpreted risk); 2) Trying to arrange an appointment within the next 4 weeks (medium interpreted risk); 3) Waiting for the next appointment in 3 months (low interpreted risk).

### Data analysis

#### Risk interpretation

To assess the effect of the presentations (Baseline, Contextualised and Grouped) on the accuracy of risk interpretation, we created a 3 × 3 confusion matrix for each presentation that reported the judgments made by patients versus our gold standard (i.e. nephrologists’ clinical judgement). From the confusion matrices, we calculated precision (i.e. proportion of correct interpretations of all interpretations as risk X), recall (i.e. proportion of correct interpretations on clinical scenarios with risk X) and accuracy (i.e. proportion of correct interpretations of all interpretations) for each presentation, and compared these using chi-squared tests.

We repeated the analysis with a secondary definition of the outcome, which aimed at investigating a situation in which, from a safety perspective, a misjudgement could have serious consequences. We evaluated the presentation’s performance in terms of patients correctly identifying the need for action (i.e. at least medium interpreted risk in medium and high risk clinical scenarios). To evaluate whether performance was driven by single patients (i.e. there were some patients that misinterpreted most of the information), we counted the mistakes that each patient made. We distinguished between: patients underestimating the need for action (i.e. low interpreted risk in medium or high risk scenarios); and patients over-estimating the need for action and asking for help when not needed (i.e. interpreted medium or high risk in low risk clinical scenarios).

#### Visual search behaviour

To investigate whether correct interpretations were related to specific visual search behaviours, our secondary objective was to evaluate differences (if any) in eye-tracking data between patients who consistently identified the need for action and those who did not in at least one occasion. We collected the following metrics:

- Fixation count: a fixation is a stable gaze on screen lasting between 40 and 500 ms. We collected the number of fixations lasting at least 180 ms [46], because higher thresholds are considered to be more reliable when the stimuli include graphical content [47]. Fixation count is an indicator of visual search efficiency [48].
- Average fixation duration: we computed the average fixation duration, as an indicator of task difficulty and cognitive load whereby longer fixation durations indicate more challenging tasks [48].
- Dwell time: this metric, which is the aggregated fixation duration, is typically an indicator of attention and interest [48].

There were three AoIs in each presentation (see Additional file 1: Figure S1): 1) tiles showing the latest values for all laboratory tests; 2) the graph showing detailed longitudinal information for a single laboratory test; 3) the graphs comparing detailed longitudinal information for two laboratory tests. At first sight, due to their clear demarcations, the natural unit for defining AoIs would have been each single tile. However, their small size would have resulted in unreliable data because the precision of the eye-tracker is compromised with smaller AoIs. What is more, all the tiles convey the same functionality so the added value of treating them independently would have been negligible. Consequently we defined larger AoIs that grouped widgets with the same appearance and functionality.

Overall differences in fixation count and dwell time were assessed with a mixed ANOVA, including patient group (i.e. patients who did not underestimate the need for action versus the others) and the within subject factors (i.e. presentation, clinical scenario and specific AoIs). We also assessed differences in fixation durations with a mixed ANOVA, this time not accounting for the specific AoI in the within-subject factors. This choice was mandated by the low frequency with which participants looked at some AoIs, therefore limiting our statistical power in assessing differences. To account for the skewedness and non-normality of residuals introduced by count and bounded data, we ran the mixed ANOVAs on the log-transformed eye fixation counts, eye fixation duration, and dwell time, rather than on the raw data.

Eye-tracking data were extracted using the Tobii Studio (version 3.4.0). All data analyses were performed in R version 3.3.1 [49].

## Results

### Patient characteristics

Table 1 reports the characteristics of the 20 patients who participated in the study. The majority were male, had at least a college education and used the internet for more than 5 h per week. Frequency of PatientView use was balanced within our study population, with 11 participants who were regular users (i.e. quarterly use) and the remaining nine using it less than twice per year.

**Table 1:** Patients’ characteristics

Parameters		Values
Number of patients		20
Gender	Female, n (%)	4 (20)
	Male, n (%)	16 (80)
Age in years, mean (SD)		51.8 (10.3)
Years since kidney transplant, mean (SD)		10.7 (8.7)
Subjective Numeracy Scale^a^, mean (SD)		4 (0.8)
Health literacy^b^, mean (SD)		0.5 (0.6)
Graph literacy^c^ score, mean (SD)		73.5 (11.3)
Education	Lower than GCSE, n (%)	1 (5)
	GCSE, n (%)	7 (35)
	A-level/College, n (%)	5 (25)
	Higher education/University degree, n (%)	7 (35)
Internet use	Less than one hour per week, n (%)	1 (5)
	One to five hours per week, n (%)	5 (25)
	Five to 10 h per week, n (%)	5 (25)
	More than 10 h per week, n (%)	9 (45)
PatientView use	Never used, n (%)	3 (15)
	No more than once per year, n (%)	5 (25)
	Twice per year, n (%)	1 (5)
	Quarterly, n (%)	11 (55)

*Abbreviations: SD* Standard deviation, *SNC* Subjective Numeracy Scale, *GCSE* General Certificate of Secondary Education

^a^On a 1–6 scale [43]

^b^On a 0–4 scale, with values close to 0 indicating better self-reported health literacy [44]

^c^Percentage [45]

### Data analysis

#### Risk interpretation

Table 2 shows the confusion matrices and performance measures (precision and recall) for all presentations across the clinical scenarios (i.e. low, medium and high risk). In most cases, patients correctly interpreted low and high risk clinical scenarios across all the presentations, while medium risk clinical scenarios were often confused with low risk ones. For each presentation, at least two patients misinterpreted high risk clinical scenarios as low risk ones. The precision scores of the interpreted risks were similar for the Baseline and Contextualised presentations, and ranged from 0.33 to 0.69 and from 0.38 to 0.70, respectively. For the Grouped presentation performance was lower, and ranged between 0.28 and 0.58. Likewise, recall scores for the Baseline and Contextualised presentations were similar, and ranged from 0.30 to 0.70. The only main difference was observed for high risk clinical scenarios, where recall was 0.70 for the Contextualised presentation and 0.55 for the Baseline. Again, a drop in performance was noticed for the Grouped presentation. Overall accuracy was 0.55 for the Contextualised presentation, 0.52 for the Baseline presentation and 0.45 for the Grouped presentation. These differences were not statistically significant, with all *P*-values in pairwise comparisons greater than 0.350.

**Table 2:** Confusion matrix of the interpreted risk by patients versus the risk of clinical scenarios as assessed by nephrologists (gold standard), and performance (i.e. precision and recall) for each presentation

Presentation	Interpreted risk by participants	Clinical scenarios (gold standard)			Performance	
		Low risk	Medium risk	High risk	Precision	Recall
Baseline	Low	14	10	2	0.54	0.70
	Medium	5	6	7	0.33	0.30
	High	1	4	11	0.69	0.55
Contextualised	Low	13	9	2	0.54	0.65
	Medium	6	6	4	0.38	0.30
	High	1	5	14	0.70	0.70
Grouped	Low	11	9	3	0.48	0.55
	Medium	7	5	6	0.28	0.25
	High	2	6	11	0.58	0.55

In addition, we calculated confusion matrices to compare situations in which an action was needed (i.e. scenarios with at least medium risk) versus those that did not require any action (i.e. low risk clinical scenarios) (see Table 3). For scenarios where an action was needed, the three presentations performed similarly with a precision around 0.8 and a recall around 0.7. However, in about 50% of the scenarios where patients interpreted that no action was needed, this was not in line with the gold standard. We did not find statistically significant differences between the different presentations, with *P*-values greater than 0.700 in pairwise comparisons.

Additional file 1: Table S1 shows the number of times patients underestimated or overestimated the need for action across all presentations and clinical scenarios. Thirteen patients (65%) underestimated the need for action at least once, of whom seven (35%) misinterpreted at least half of the scenarios requiring an action. Fourteen patients (70%) overestimated the need for action at least once. Individual patient characteristics are reported in Additional file 1: Table S2 and S3.

**Table 3:** Confusion matrix of the interpreted risk by patients versus the gold standard (nephrologists judgement) and performance (i.e. precision and recall) for clinical scenarios where an action was needed (i.e. at least medium risk) and those where no action was needed (i.e. low risk)

Presentation	Interpreted risk by participants	Clinical scenarios (gold standard)		Performance	
		Action needed	No action needed	Precision	Recall
Baseline	Action needed	28	6	0.82	0.70
	No action needed	12	14	0.54	0.70
Contextualised	Action needed	29	7	0.81	0.72
	No action needed	11	13	0.54	0.65
Grouped	Action needed	28	9	0.76	0.70
	No action needed	12	11	0.48	0.55

#### Visual search behaviour

We were not able to track eye movements for two participants, resulting in 18 patients being included in the eye tracking data analysis. Encountering difficult eye-tracking circumstances in about 10% of participants is not uncommon in eye tracking studies [50]. This can be related to participant’s characteristics like glasses, particular shapes of eye-lids or very small pupils [50].

Overall, we observed 5071 eye fixations across all patients and clinical tasks on any of the three AoIs, with an overall time of 1416 s (23.6 min) and a mean fixation duration of 0.279 s (Standard Deviation [SD], 0.129 s). The median total eye fixation count per patient across the nine tasks was 255 (Inter Quartile Interval [IQI], 108–422), while the median total dwell time was 75 s (IQI, 29–120 s).

**Fig. 2:**
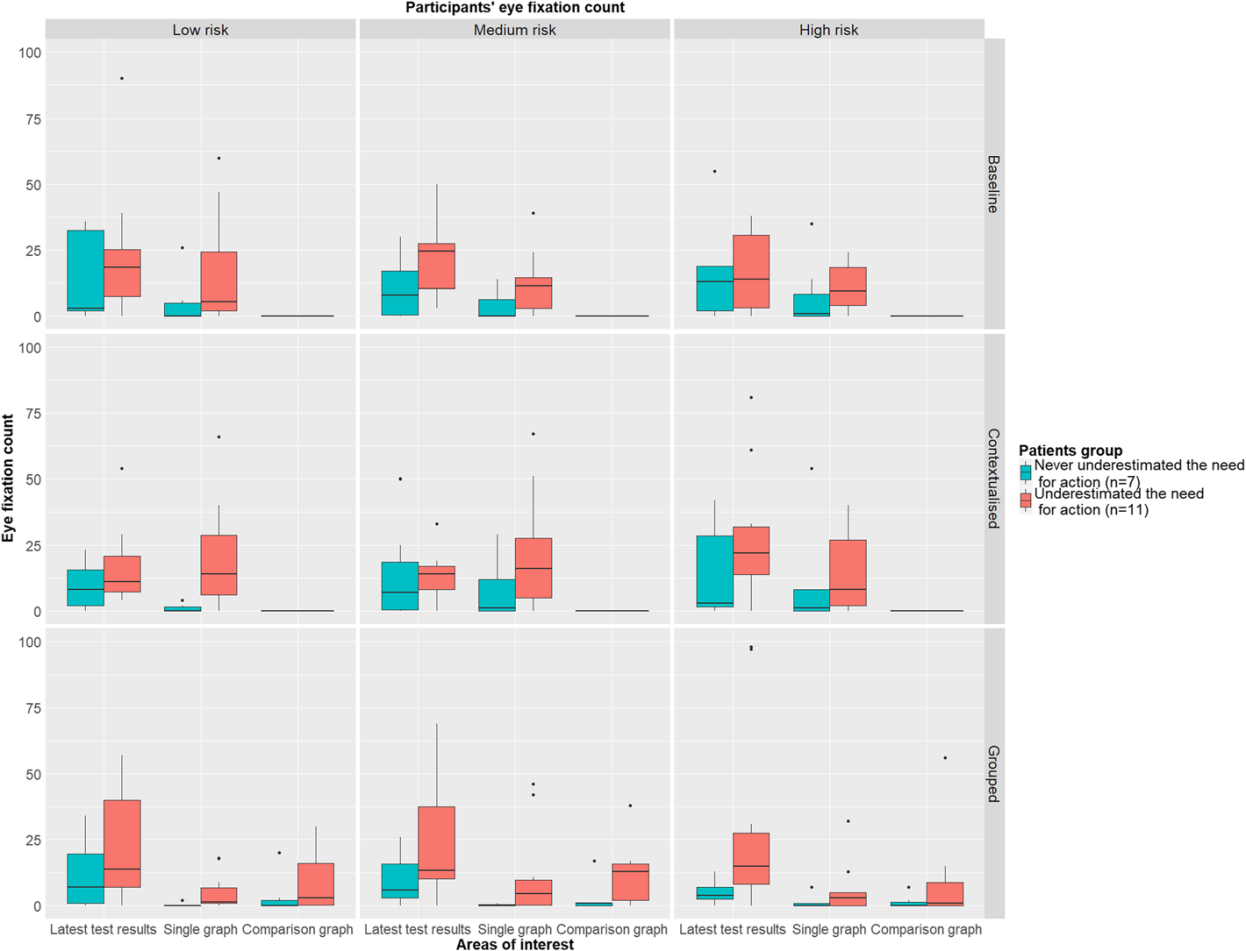
Fixation count on the different areas of interest across the presentations and clinical scenarios for patients who never underestimated the need for action (*n* = 7) and those who did (*n* = 11). To facilitate comprehension four measurements pertaining to the 99th percentile (i.e. fixation count greater than 100) were excluded from this graph

Of the patients for whom we could track eye movements, eleven underestimated the need for action at least once, while seven consistently understood when an action was needed. Figure 2 shows their eye fixation counts across the different presentations, scenarios, and AOIs, with medians ranging between 0 and 25 eye fixations. No statistically significant differences were found among the different presentations in the mixed ANOVA (*P* = 0.247), with median fixation count that was 22 (IQI, 6–46), 20 (IQI, 8–43), and 21 (IQI, 6–44) for the Baseline, Contextualised and Grouped presentation, respectively. Also no significant differences were found between the different clinical scenarios (*P* = 0.842), for which we observed an overall median of 18 (IQI, 7–48) fixations for low risk clinical scenarios, 23 (IQI, 6–39) fixations for medium risk clinical scenarios, and 21 (IQI, 5–45) fixations for high risk clinical scenarios. Conversely, different patterns were found for the different AoIs, with a *P*-value below 0.001 in the mixed ANOVA. Particularly, patients mostly looked at the AoI containing the summary of the latest test results, with a median of 13 fixations (IQI, 4–26) against 3 (IQI, 0–13) and 0 (IQI, 0–0) for the single graph and comparison graphs AoIs respectively. The latter was mainly looked at in the Grouped presentation. This was confirmed by the mixed ANOVA test that found a statistically significant result for the interaction term between presentation and the specific AoI (*P* < 0.001). In terms of the main difference between the two groups of patients, we found that patients who never underestimated the need for action required fewer eye fixations to make a decision, with overall median fixation counts of 11 (IQI, 2–28) and 32 (IQI, 13–58). This was shown also by the mixed ANOVA, where the term referring to patient’s group was statistically significant (*P* = 0.021). Similar results were found for dwell time (see Additional file 1: Figure S10).

**Fig. 3:**
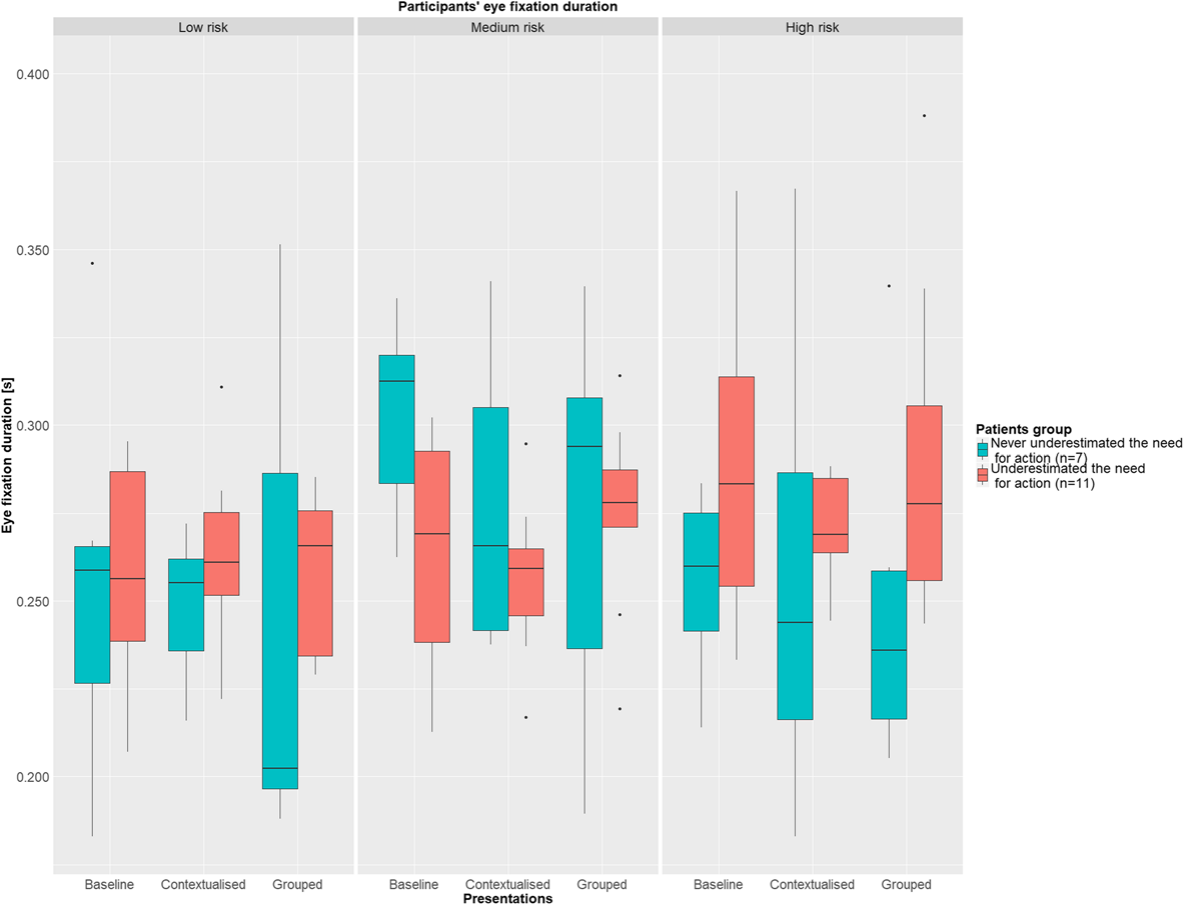
Fixation duration (in seconds) across the three different clinical scenarios and three different presentations. Participants are stratified by those who never underestimated the need for action (*n* = 7) and those who did (*n* = 11). To facilitate comprehension two measurements pertaining to the 99th percentile (i.e. fixation durations greater than 0.400 s) were excluded from this graph

Eye fixation durations are shown in Fig. 3, with medians between 0.202 and 0.313 s. We did not find differences in eye fixation durations between the different presentations (*P* = 0.186), which had median fixation duration values between 0.260 (IQI, 0.245–0.275) seconds for the Contextualised presentation and 0.268 (IQI, 0.243–0.294) for the Baseline presentation. Also no difference was found across patient groups for the different tasks (*P* = 0.357), with a median of 0.268 (IQI, 0.246–0.287) seconds for patients who underestimated the need for action and 0.259 (IQI, 0.218–0.303) seconds for those who did not. The only statistically significant difference we found was between low, medium, and high risk clinical scenarios (*P* = 0.028). Median values were 0.255 (IQI, 0.231–0.275), 0.272 (IQI, 0.246–0.298) and 0.266 (IQI, 0.246–0.289) seconds, respectively. This is not surprising, as lower eye fixation durations reflect less cognitive demand, which is expected for low risk clinical scenarios. Although not statistically significant (P of interaction term equal to 0.161), it is interesting to highlight how the two groups of patients showed different cognitive loads in relation to the risk presented. Particularly, for low and high risk clinical scenarios, patients who did not underestimate the risk had lower median values across the three presentations. While the opposite was observed for medium risk clinical scenarios.

## Discussion

We conducted a controlled study to investigate whether presentations using colours and graphical cues (e.g., horizontal coloured bars and personalised grouping) improve patients’ abilities to correctly interpret laboratory test results presented through patient portals.

We found no influence of presentation on the accuracy of risk interpretation, with misinterpretation of risk being consistently high across the three presentations. Notably, 65% of patients underestimated the need for action at least once, and in 50% of the scenarios where patients decided not to act on the presented information it would have been better to do so. This happened despite the presentation of visual and graphical cues; grouping of laboratory test results with normal and abnormal readings; and personalised descriptive statistics. These findings raise patient safety concerns and confirms that the presentation of laboratory test results, in terms of interface design alone, might not be enough to ensure the accuracy of risk interpretation [19].

To the best of our knowledge this is the first study to assess the effect of different presentations of laboratory test results on risk interpretation by implementing presentations of different systems where patients could explore a broad panel of laboratory results and detailed longitudinal information. Previous studies have examined how patients interpret risk in the context of laboratory test results [15–17, 23, 51]. While these studies also found that patients had difficulties in interpreting risk correctly, only two made comparisons between different presentations of laboratory test results [16, 23]. Particularly, Brewer et al. [16] and Zikmund-Fisher et al. [23] compared a tabular format to horizontal coloured bars. In line with our results, Brewer et al. [16] did not find significant improvement in recall. Conversely, Zikmund-Fisher et al. [23] found improvements in terms of understanding the level of urgency of near-normal laboratory test results when using coloured bars instead of a tabular format. They also found that high risk clinical scenarios were perceived as urgent, independent of the type of presentation used, which is consistent with our findings. Their finding on near-normal results is difficult to compare to our results as we did not test this clinical scenario: conversely to our medium risk clinical scenarios, their aim was to decrease the level of perceived urgency rather than prompt an action.

As shown by the confusion matrix in Table 2, participants in our study found it more difficult to recognise medium risk than low or high risk clinical scenarios. In addition, as displayed by the eye tracking data, medium risk clinical scenarios seemed to be more cognitively demanding even for those who never underestimated the need for action. This is a unique finding of our study, as most of previous studies that looked at patients’ interpretation of laboratory test results limited the task to the identification of abnormal values [15, 16, 52]. We used an approach similar to Zikmund-Fisher et al. [17, 23] who evaluated participants’ behavioural intentions in relation to interpretation of laboratory test results. The difference in our study was the explicit use of participant’s behavioural intentions as a proxy of their interpreted risk. This allowed us to reflect different clinical scenarios that are likely to happen in real life, and evaluate patients’ interpretation in a more naturalistic way.

This is the first study to adopt eye-tracking data in the context of risk interpretation of laboratory test results in patient portals. We found that patients who never underestimated the need for action had less eye-fixation counts and a shorter dwell time across the different presentations and clinical scenarios. This suggests a more targeted visual search behaviour consistently adopted by these patients, who were able to filter the data on-screen and focus their attention on the relevant pieces of information. These pieces of information are likely to be the laboratory tests most relevant to their condition, helping them to understand whether an action was needed. Conversely, patients who underestimated the need for action distributed their attention over the different AoIs for longer dwell time and more eye-fixation counts suggesting difficulty in finding the right piece of information to focus on. The importance of these results are twofold. First, this confirms the importance of patient education (i.e. teaching which laboratory tests are more important and when they should act) prior to the use of patient portals [53]. Second, from an interface design perspective, further highlighting the pieces of information that are most relevant for patients and filtering those that are less relevant might enhance patients’ interpretation. Without making dramatic changes to the PatientView interface we used for our study, this could be achieved by taking advantage of reading patterns on the Web and placing the most relevant information accordingly: attention decreases from left to right and from the top to the bottom area of the screen following an F-shaped reading pattern [54]. Therefore, placing relevant information closer to the left-top corner would remove information overload and facilitate decision-making. If the layout of the user interface could be altered, the most relevant information could be allocated a larger area than the remaining pieces of information shown (e.g. larger tiles if we refer to the PatientView interface), as size of the user interface elements has been proved to be one of the main features to draw attention on Web pages [55].

### Limitations

Our study has several limitations. First, since our objective was to study risk interpretation and visual search behaviour in depth, each participant had to visit our eye-tracking laboratory, requiring a substantial time and financial investment from the research team. Due to research resource limitations, it was not possible to perform the study with a larger cohort of patients. This limited our statistical power to investigate the association between risk interpretation and patient characteristics (i.e. health literacy, numeracy and graph literacy), which have previously been shown to be relevant in the context of laboratory test result interpretation [17, 23]. Furthermore, although the population we selected was homogeneous in terms of their experience with monitoring their laboratory test results (i.e. all having quarterly tests after the kidney transplant), the small sample size limits the generalisability of our findings. However, our study still provides important insight on the risk interpretation and visual search behaviours of patients accessing laboratory test results on patient portals, and it should be considered as complementary, rather than alternative, to other studies that had larger populations, but studied risk interpretation in less depth (e.g. using static prototypes or surveys). Second, patients were given fictitious clinical scenarios to assess risk, in which they might have been less cautious because of the simulation nature of the study. Observing patients’ interpretation of data from their own laboratory test results could simulate a more naturalistic research environment and strengthen the applicability of our results in clinical practice. Finally, since the study was performed in a laboratory setting where participants knew they were observed and studied, participants’ behaviours might have been different than their behaviours in real life as consequence (i.e. Hawthorne effect).

## Conclusions

This study confirmed patients’ difficulties in interpreting laboratory test results, with many patients underestimating the need for action even when abnormal values were highlighted using colours and graphical cues. Participants who consistently understood when action was needed showed a higher visual search efficiency, suggesting a better strategy to cope with information overload, enabling them to focus on the laboratory tests most relevant to their condition. Our findings raise concerns over the limitations of patient portals in supporting self-care and possible safety risks.

## Additional file

Additional file 1: **Figures S1-S9** that show details of the three Presentations, highlighting the three areas of interest under study; **Table S1** that reports a contingency table for the number of patients who underestimated and overestimated the need for action; **Tables S2** and **S3** that show the characteristics of patients who underestimated and overestimated the need for actions and those who did not; **Figure S10** that shows participant’s dwell time on the different areas of interest across the different presentations and clinical scenarios, stratified by patients who underestimated the need for action and those who did not. (DOCX 2455 kb)

## Abbreviations

AoI: Area of interest
CKD: Chronic Kidney Disease
eGFR: estimated Glomerular Filtration Rate
IQI: Interquartile interval
SD: Standard deviation
SNC: Subjective Numeracy Scale
SRFT: Salford Royal NHS Foundation Trust
