# Supplementary material for: Presentation of laboratory test results in patient portals: influence of interface design on risk interpretation and visual search behaviour
Source: BMC Med Inform Decis Mak. 2018 Feb 12;18:11. doi: 10.1186/s12911-018-0589-7 (PMC5809992; doi:10.1186/s12911-018-0589-7)

**Additional file 1**

**Figure S1:** Latest test results for the Baseline presentation. The yellow rectangle delimits the related Area of Interest used in the eye-tracking analysis.

**
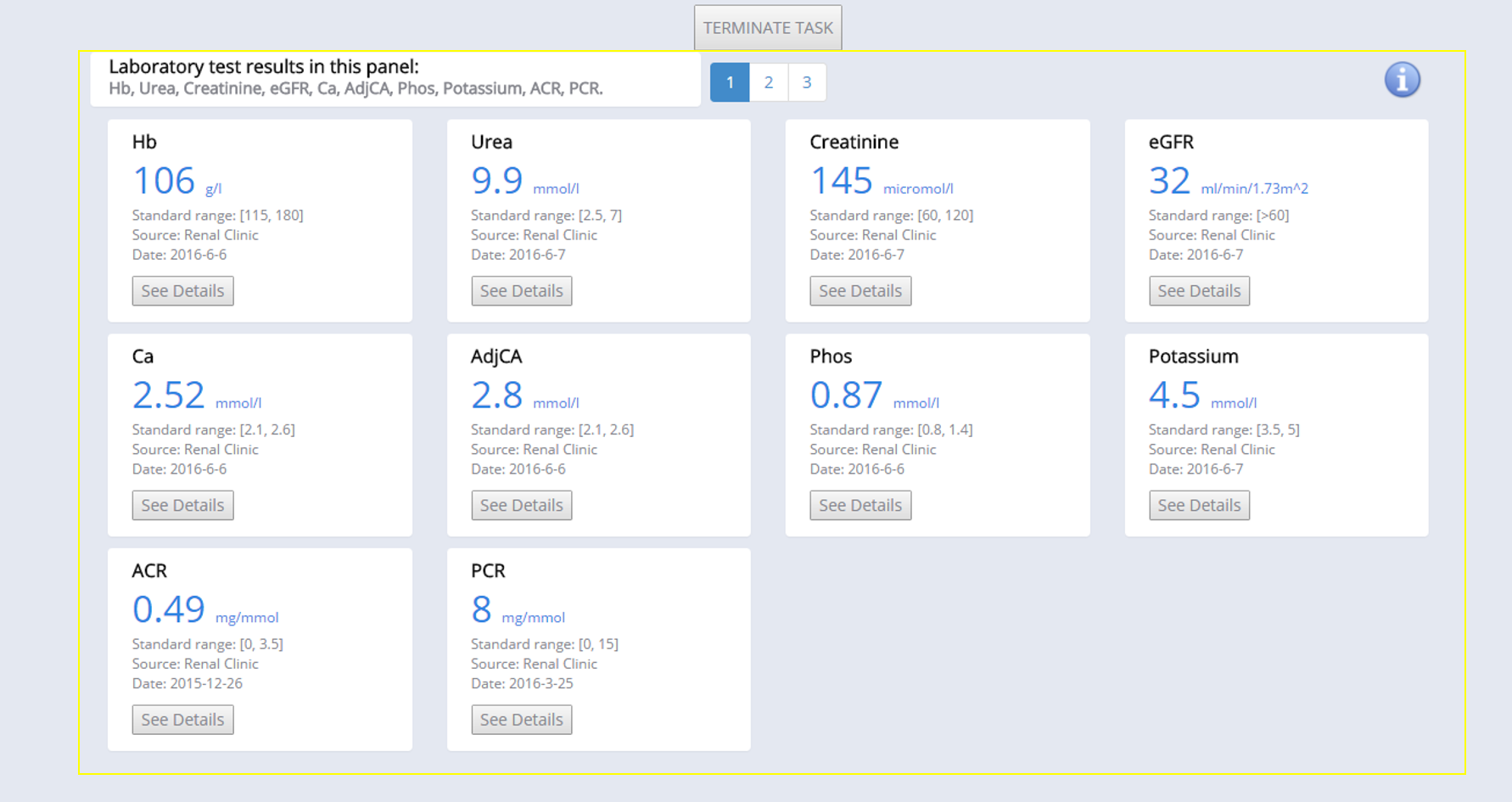
**

**Figure S2:** Graph showing longitudinal information pertaining to a single parameter for the Baseline presentation. The blue rectangle delimits the related Area of Interest used in the eye-tracking analysis.


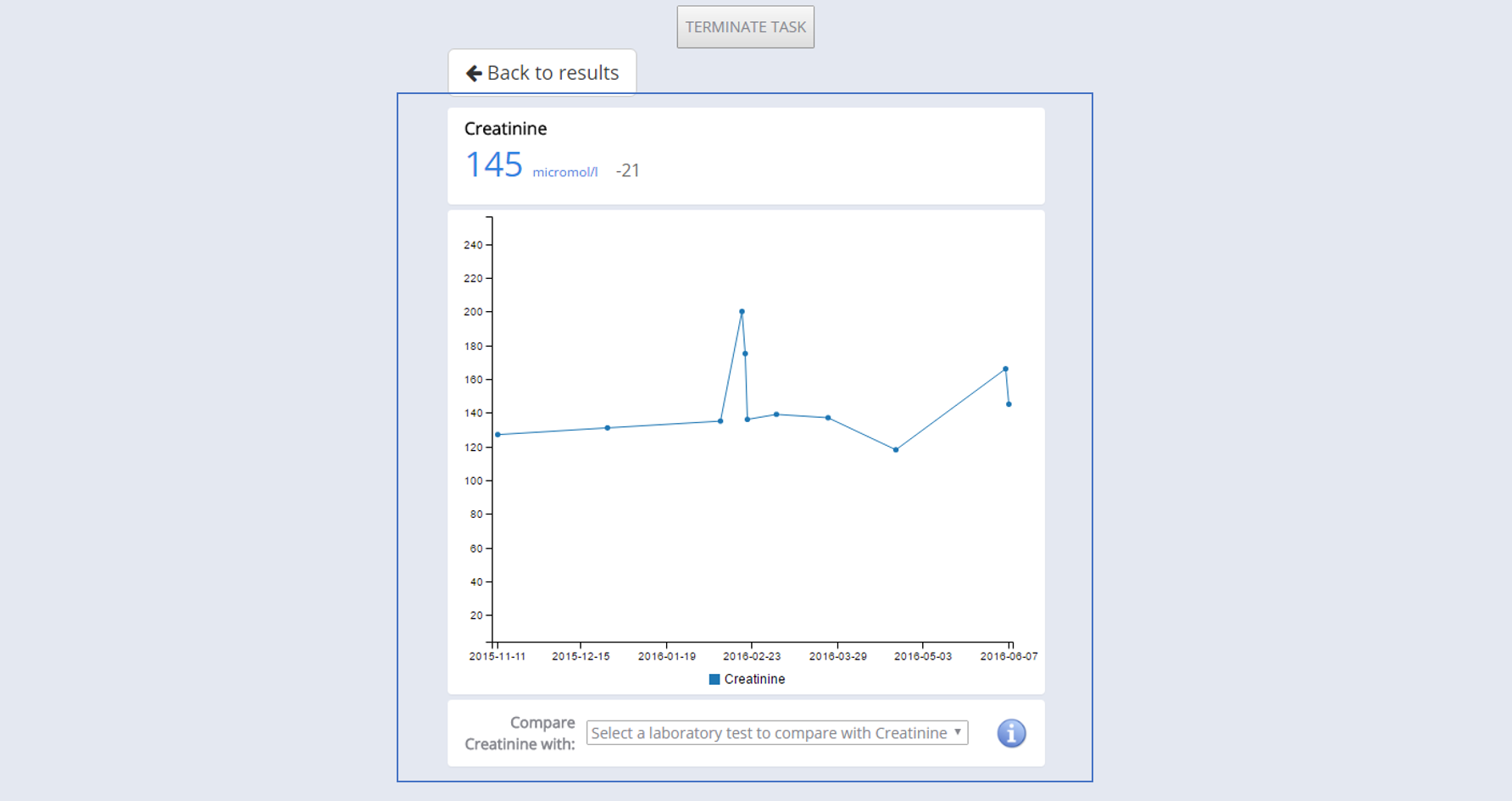


**Figure S3:** Graph comparing longitudinal information pertaining to two parameters at the same time for the Baseline. The purple rectangle delimits the related Area of Interest used in the eye-tracking analysis.

**
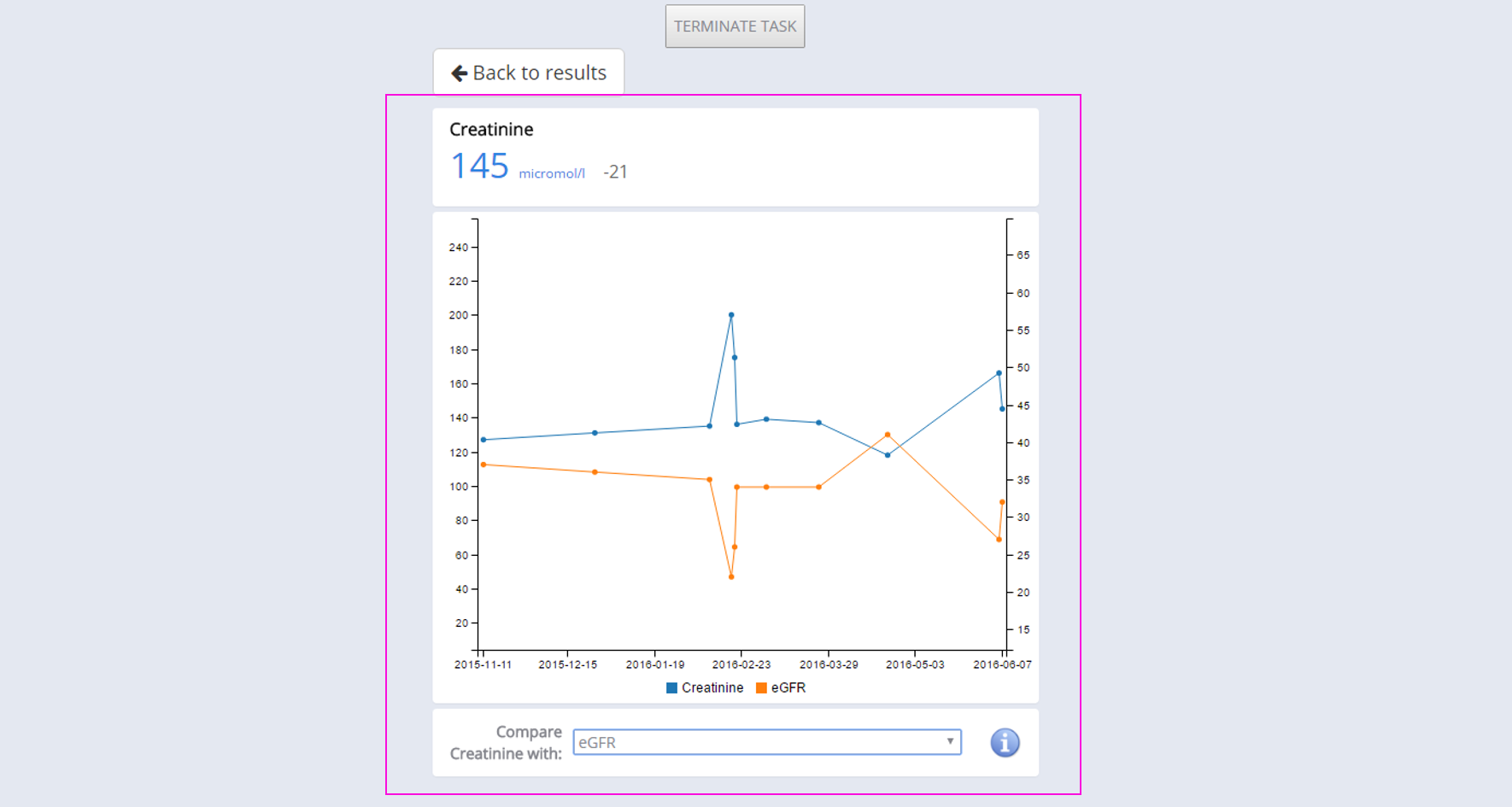
**

**Figure S4:** Latest test results for the Contextualised presentation. The yellow rectangle delimits the related Area of Interest used in the eye-tracking analysis.

**
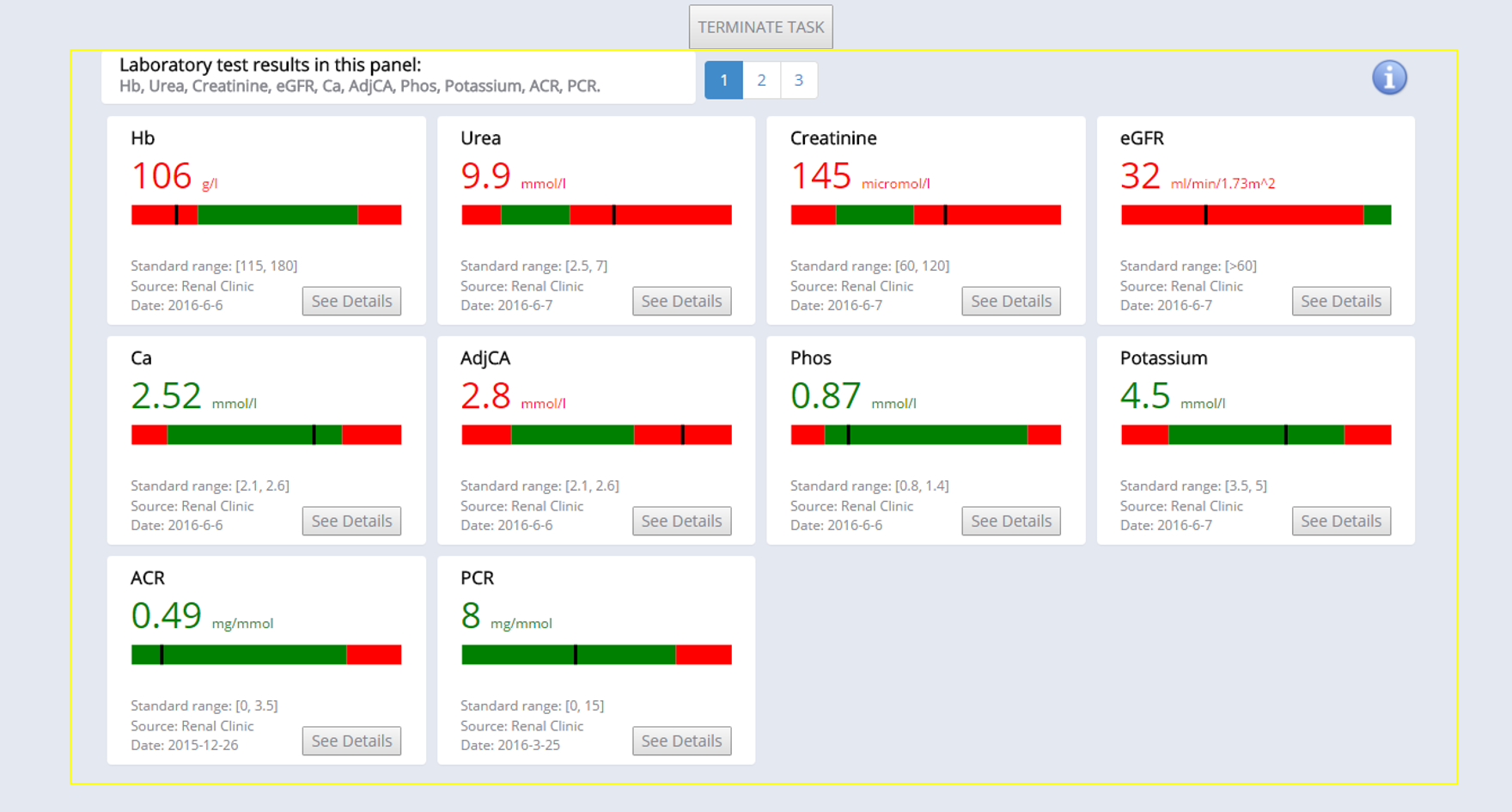
**

**Figure S5:** Graph showing longitudinal information pertaining to a single parameter for the Contextualised presentation. The blue rectangle delimits the related Area of Interest used in the eye-tracking analysis.


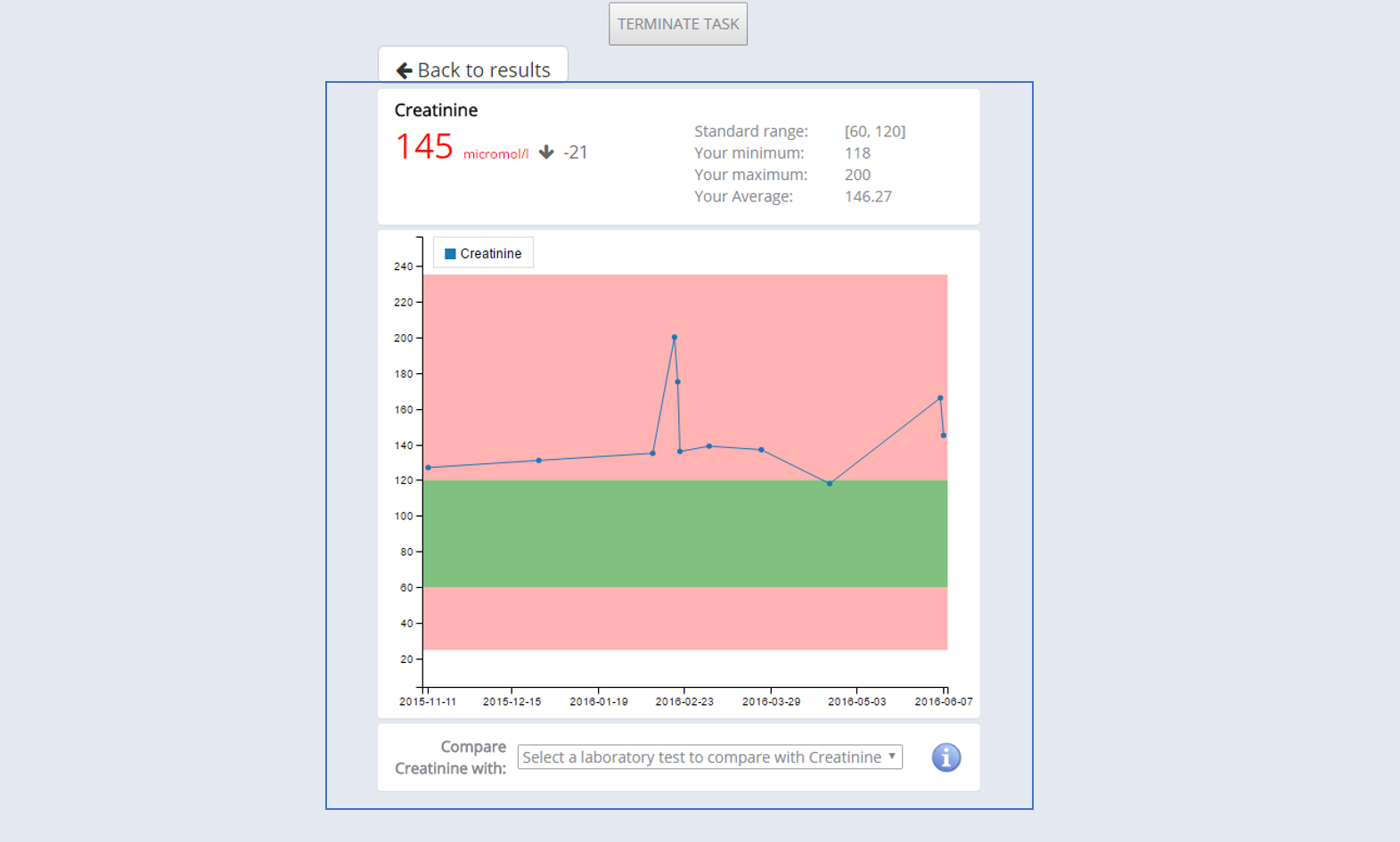


**Figure S6:** Graphs comparing longitudinal information pertaining to two parameters at the same time for the Contextualised. The purple rectangle delimits the related Area of Interest used in the eye-tracking analysis.

**
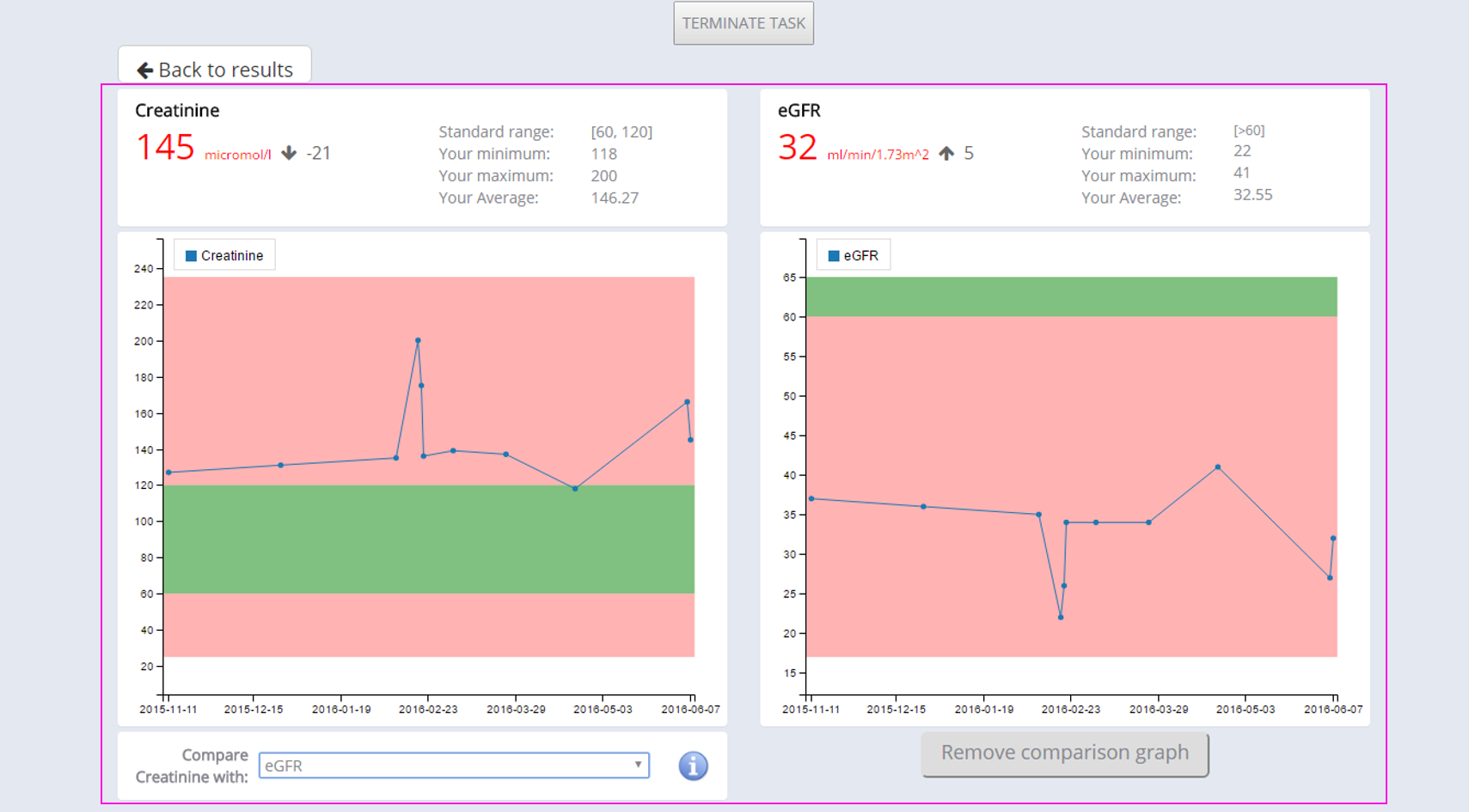
**

**Figure S7:** Latest test results for the Grouped presentation. The yellow rectangle delimits the related Area of Interest used in the eye-tracking analysis.

**
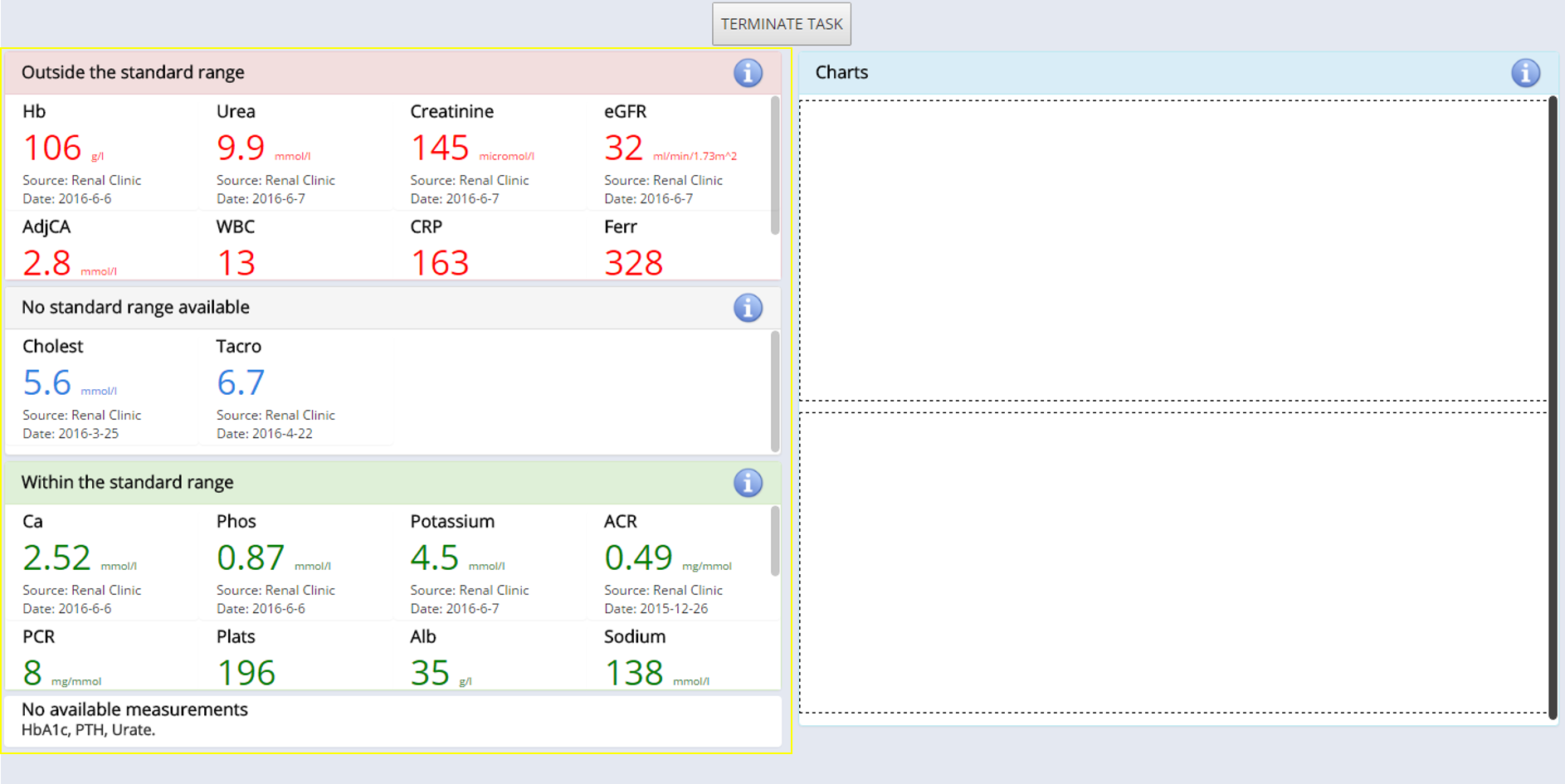
**

**Figure S8:** Latest test results and graph showing longitudinal information pertaining to a single parameter for the Grouped presentation. The yellow and blue rectangle delimit the related Area of Interest used in the eye-tracking analysis.


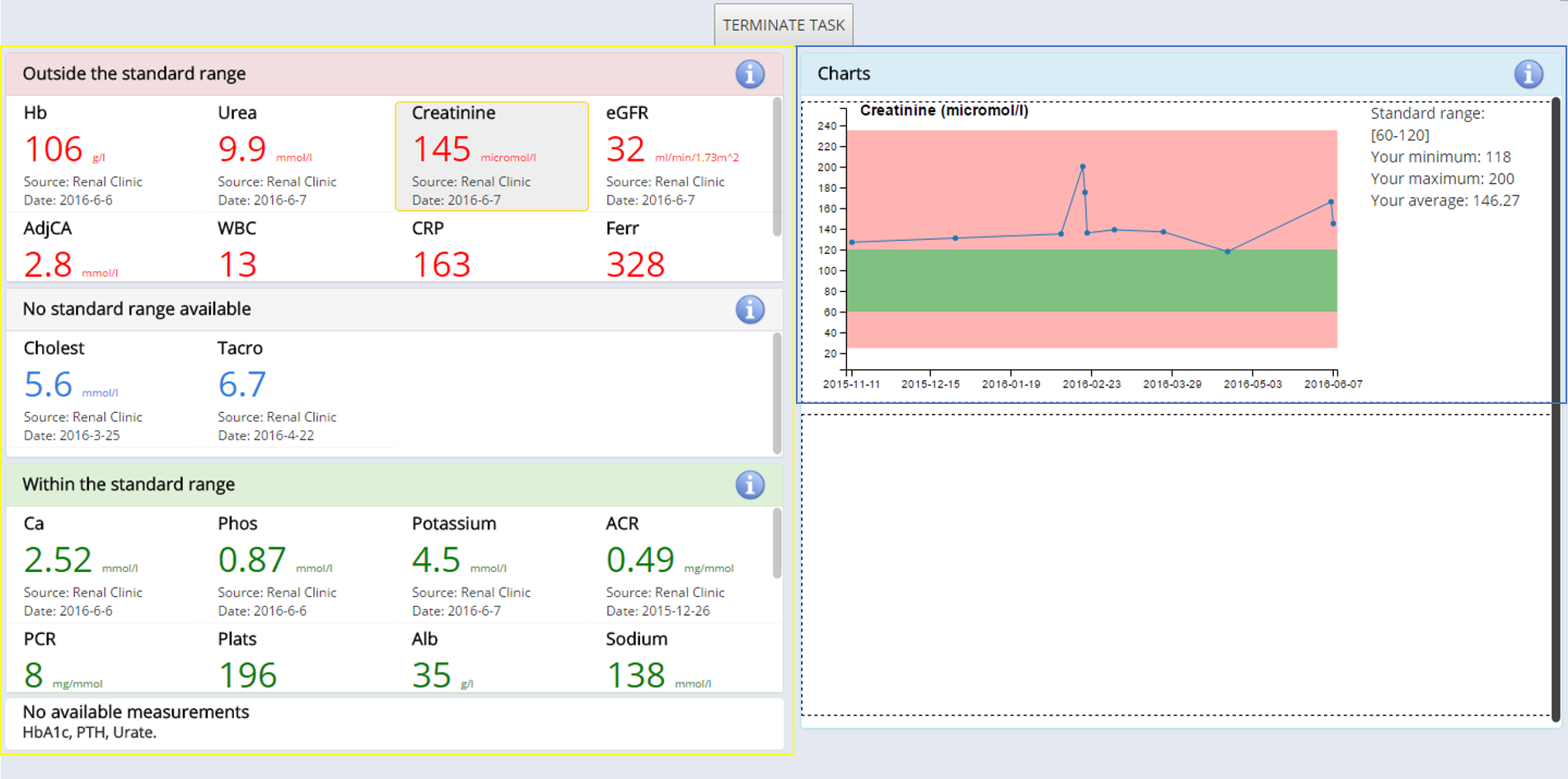


**Figure S9:** Latest test results and graphs comparing longitudinal information pertaining to two parameters for the Grouped presentation. The yellow and purple rectangle delimit the related Area of Interest used in the eye-tracking analysis.

**
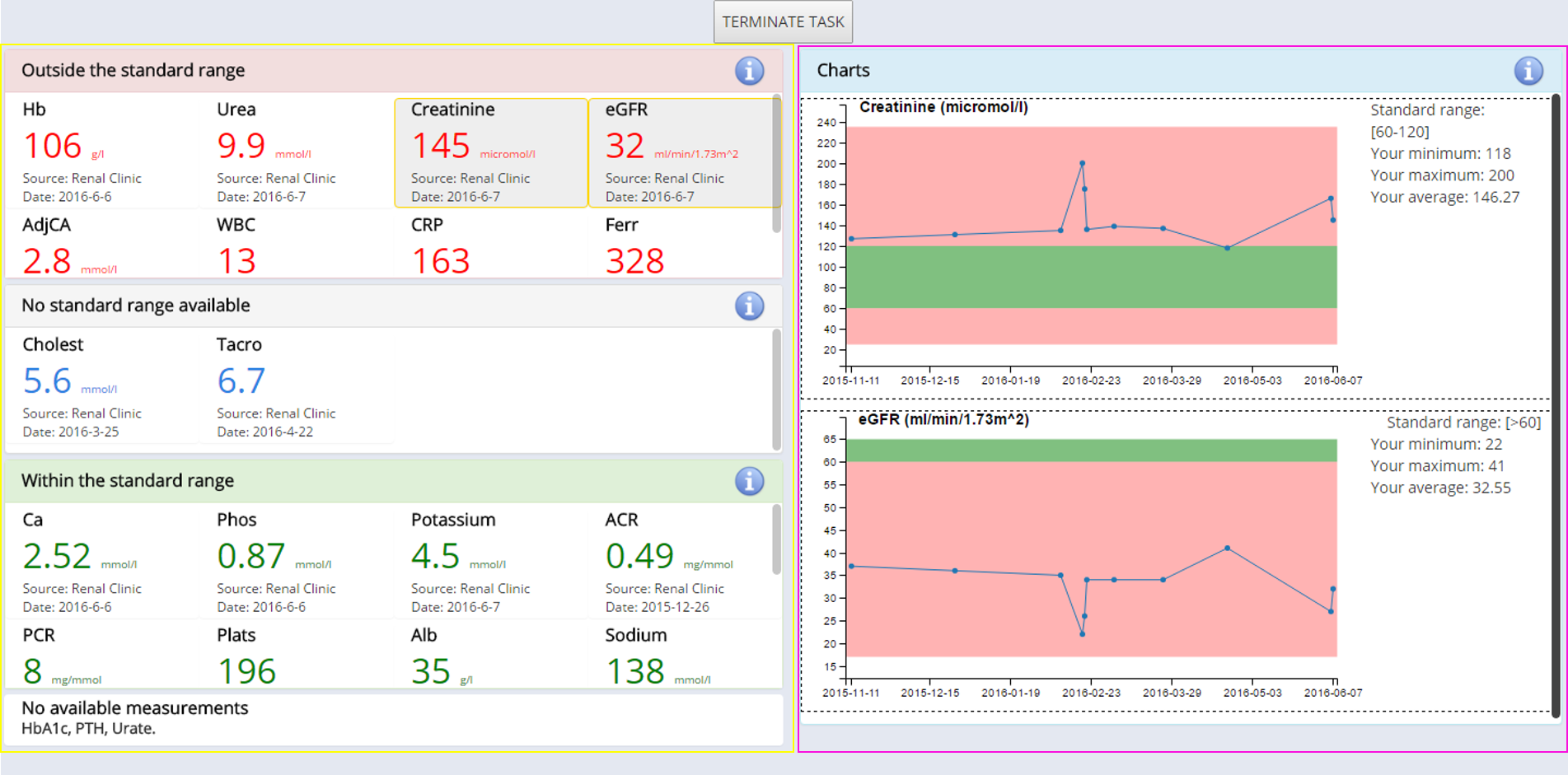
**

**Table S1:** Contingency table for the number of patients who underestimated and overestimated the need for action.

| **Number of times** | **Number of patients (n=20)** | |
| --- | --- | --- |
|  | **Underestimating the**  **need for action (%)** | **Overestimated the**  **need for action (%)** |
| 0 | 7 (35) | 6 (30) |
| 1 | 3 (15) | 8 (40) |
| 2 | 3 (15) | 4 (20) |
| 3 | 4 (20) | 2 (10) |
| 4 | 2 (10) | / |
| 5 | 0 (0) | / |
| 6 | 1 (5) | / |

**Table S2:** Characteristics of patients who underestimated the need for action and those who did not. Abbreviations: SD, Standard deviation; GCSE: General Certificate of Secondary Education.

| **Parameters** | | **Patients who did not**  **underestimate the need for action** | **Patients who underestimated the need for action** |
| --- | --- | --- | --- |
| Number of patients | | 7 | 13 |
| Gender | Female (%) | 1 (14) | 3 (23) |
|  | Male (%) | 6 (86) | 10 (77) |
| Age (years) | | 51.1 (12.9) | 52.2 (9.1) |
| Years since kidney transplant (mean, SD) | | 11.9 (9.6) | 10 (8.5) |
| Subjective Numeracy Scale ^a)^ score (mean, SD) | | 3.7 (0.9) | 4.1 (0.9) |
| Health literacy ^b)^ (mean, SD) | | 0.3 (0.3) | 0.6 (0.7) |
| Graph literacy score ^c)^ (mean, SD) | | 72.5 (11.6) | 74.5 (11.1) |
| Education | Lower than GCSE (%) | 1 (14) | 0 (0) |
|  | GCSE (%) | 1 (14) | 6 (46) |
|  | A-level/College (%) | 2 (29) | 3 (23) |
|  | Higher education/University degree (%) | 3 (43) | 4 (31) |
| Internet use | Less than one hour per week (%) | 0 (0) | 1 (8) |
|  | One to five hours per week (%) | 1 (14) | 4 (31) |
|  | Five to 10 hours per week (%) | 2 (29) | 3 (23) |
|  | More than 10 hours per week (%) | 4 (57) | 5 (38) |
| PatientView use | Never used (%) | 1 (14) | 2 (15) |
|  | Less or equal than once per year (%) | 1 (14) | 4 (31) |
|  | Twice per year (%) | 0 | 1 (8) |
|  | Quarterly (%) | 5 (71) | 6 (46) |

1. On a 1-6 scale [40]
2. On a 0-4 scale, with values close to 0 indicating better self-reported health literacy [41]**.**
3. Percentage [42].

**Table S3:** Characteristics of patients overestimated the need for action and those who did not. Abbreviations: SD, Standard deviation; SNC: Subjective Numeracy Scale; GCSE: General Certificate of Secondary Education.

| **Parameters** | | **Patients who did not overestimate the**  **need for action** | **Patients who overestimated the**  **need for action** |
| --- | --- | --- | --- |
| Number of patients | | 6 | 14 |
| Gender | Female (%) | 3 (50) | 1 (7) |
|  | Male (%) | 3 (50) | 13 (93) |
| Age (years) | | 52.8 (5.8) | 51.4 (11.8) |
| Years since kidney transplant (mean, SD) | | 15.5 (9.9) | 8.6 (7.5) |
| Subjective Numeracy Scale score ^a)^ (mean, SD) | | 3.8 (0.8) | 4.1 (0.8) |
| Health literacy ^b)^ (mean, SD) | | 0.7 (0.4) | 0.4 (0.6) |
| Graph literacy score ^c)^ (mean, SD) | | 71.8 (10.5) | 74.7 (11.5) |
| Education | Lower than GCSE (%) | 1 (17) | 0 (0) |
|  | GCSE (%) | 3 (50) | 4 (29) |
|  | A-level/College (%) | 1 (17) | 4 (29) |
|  | Higher education/University degree (%) | 1 (17) | 6 (43) |
| Internet use | Less than one hour per week (%) | 0 (0) | 1 (7) |
|  | One to five hours per week (%) | 3 (50) | 2 (14) |
|  | Five to 10 hours per week (%) | 1 (17) | 4 (29) |
|  | More than 10 hours per week (%) | 2 (33) | 7 (50) |
| PatientView use | Never used (%) | 1 (17) | 2 (14) |
|  | Less or equal than once per year (%) | 0 (0) | 5 (36) |
|  | Twice per year (%) | 0 (0) | 1 (7) |
|  | Quarterly (%) | 5 (83) | 6 (43) |

1. On a 1-6 scale [40]
2. On a 0-4 scale, with lower values indicating better self-reported health literacy [41]**.**
3. Percentage [42].

**Figure S10:** Participant’s dwell time on the different areas of interest across the different presentations and clinical scenarios for patients who underestimated the need for action (n=11) and those who did not (n=7).


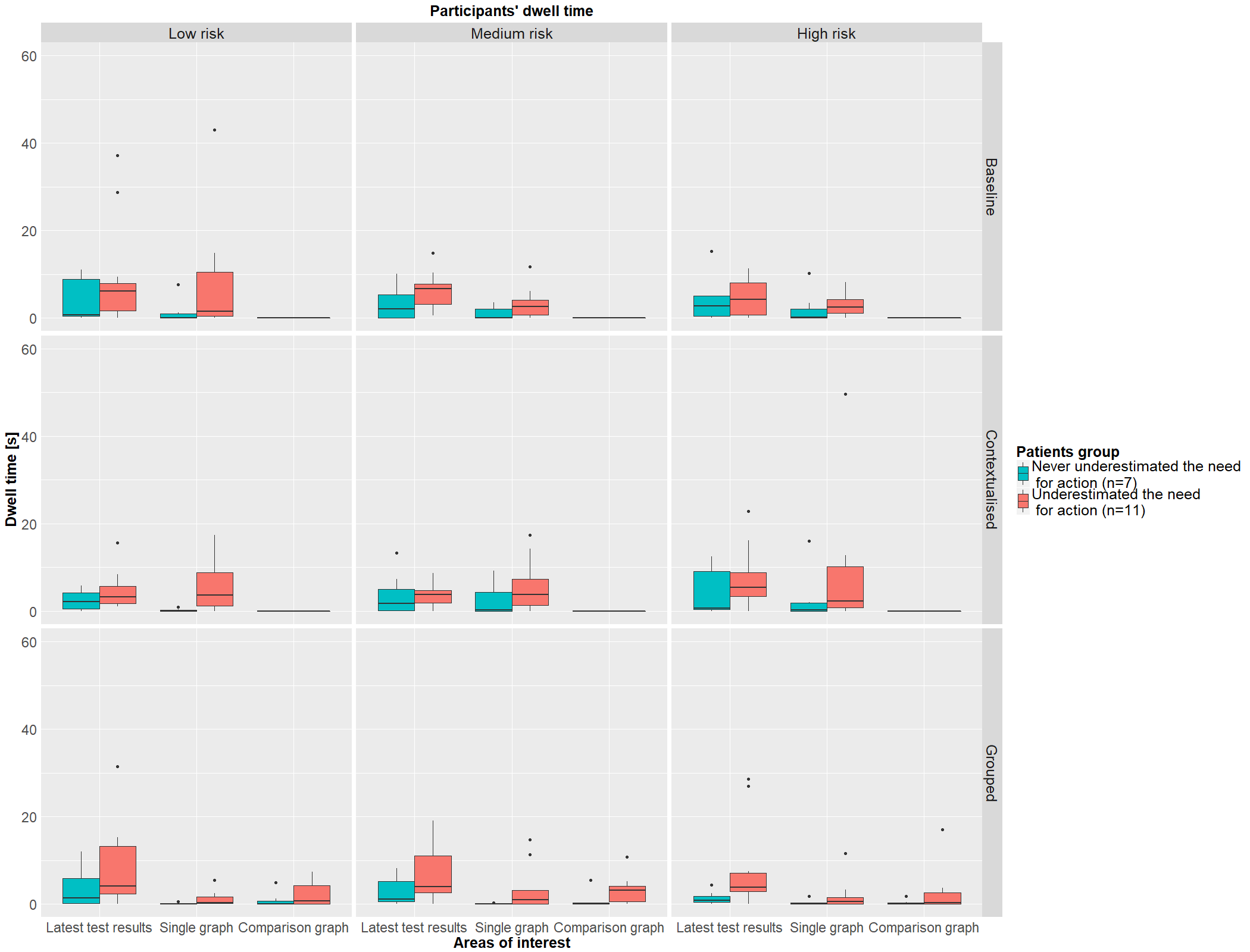

Supplement: Supplementary file 1 — Figures S1-S9 that show details of the three Presentations, highlighting the three areas of interest under study; Table S1 that reports a contingency table for the number of patients who underestimated and overestimated the need for action; Tables S2 and S3 that show the characteristics of patients who underestimated and overestimated the need for actions and those who did not; Figure S10 that shows participant’s dwell time on the different areas of interest across the different presentations and clinical scenarios, stratified by patients who underestimated the need for action and those who did not. (DOCX 2455 kb) [file 12911_2018_589_MOESM1_ESM.docx]
